# Facilitated Versus Self-Directed Educational Modalities in Palliative Care Training: A Randomized Controlled Trial of the CAPACITI Intervention

**DOI:** 10.1089/pmr.2025.0010

**Published:** 2025-06-17

**Authors:** Midori Matthew, Daryl Bainbridge, Jeff Myers, Oren Levine, Leah Steinberg, Nadia Incardona, Samantha Winemaker, Kathy Kortes-Miller, Kelli Stajduhar, Frances Kilbertus, Jose Pereira, Hsien Seow

**Affiliations:** ^1^ Faculty of Health Sciences, McMaster University, Hamilton, Ontario, Canada.; ^2^ Department of Oncology, McMaster University, Hamilton, Ontario, Canada.; ^3^ Division of Palliative Care, University of Toronto, Toronto, Ontario, Canada.; ^4^ Department of Family and Community Medicine, University of Toronto, Toronto, Ontario, Canada.; ^5^ Department of Social Work, Lakehead University, Thunder Bay, Ontario, Canada.; ^6^ School of Nursing, University of Victoria, Victoria, British Columbia, Canada.; ^7^ Northern Ontario School of Medicine University, Sudbury, Ontario, Canada.; ^8^ Department of Family Medicine, McMaster University, Hamilton, Ontario, Canada.; ^9^ The Institute for Culture and Society, University of Navarra, Pamplona, Spain.

**Keywords:** education, health personnel, palliative care, randomized controlled trial, training programs.

## Abstract

**Background:**

Community Access to Palliative Care via Interprofessional Teams Improvement (CAPACITI) is a virtual educational program designed to support primary care providers in delivering a palliative approach to care. This study evaluated whether expert-facilitated sessions result in greater self-rated competency than a self-directed format across three CAPACITI modules: Identification and Assessment, Communication, and Ongoing Management.

**Methods:**

We conducted a randomized controlled trial where 566 interprofessional primary care team members were randomized to facilitated or self-directed delivery of the CAPACITI program. Participants completed two validated surveys at baseline and after each module: the End-of-Life Professional Caregiver Survey and the CAPACITI Competencies survey. These assessed self-rated comfort and competency in providing palliative care.

**Results:**

Of the 566 participants randomized, 378 completed Module 1, and 270 and 192 completed Modules 2 and 3, respectively. Participants in both study arms demonstrated significant improvements in self-assessed comfort and competency following each module. No significant differences were observed between the facilitated and self-directed groups across either survey instrument.

**Conclusion:**

Participants in both the facilitated and self-directed study arms reported significant increases in self-rated comfort and competency in providing a palliative approach to care. CAPACITI demonstrates that a relatively large, intensive, and feasible training program can be implemented virtually across diverse care settings. These results support the broader application of structured, scalable educational interventions in primary care, particularly those grounded in practical content and adult learning principles.

**Trial registration number:**

ClinicalTrials.gov NCT05120154. Date registered: Oct 15, 2021. The authors affirm that this trial was registered prior to enrolling any participants.

## Introduction

As populations live longer with greater chronic disease burden, there is an increasing need for early palliative care approach for individuals experiencing life-limiting illness.^1,2^ Primary care providers are well-positioned to initiate palliative care due to their long-standing patient relationships.^3,4^ However, barriers such as limited training, confidence, and practical strategies hinder their ability to deliver palliative care.^5,6^ Educational programs like Pallium Canada’s LEAP,^7^ and the UK’s Gold Standards Framework,^8^ often use in-person, facilitated workshops to enhance learning.^9^ While online programs address geographic and workforce barriers, they may face technological challenges and reduced learner appeal.^10,11^

E-learning has supported palliative care education for over two decades, with adaptations accelerating during the COVID-19 pandemic.^12–15^ The effectiveness of in-person, virtual, or hybrid delivery depends on the learning objectives and adult learning principles applied.^16,17^ Virtual and hybrid approaches show similar efficacy to classroom-based learning,^7,18^ but high-quality evidence comparing facilitated and self-directed virtual programs is limited.^16,19^

We developed Community Access to Palliative Care via Interprofessional Teams Improvement (CAPACITI), a virtual program offering practical strategies for primary care teams to adopt a palliative approach.^17,20,21^ A pilot study showed improved palliative care identification and competency outcomes in a single cohort pre-post evaluation.^17,21^ We revised CAPACITI based on quantitative and qualitative^21^ findings, reorganizing the content into three distinct modules, and conducted a cluster randomized controlled trial (RCT) of this program comparing facilitated versus self-directed modalities. We previously reported on the effectiveness of these two study arms on improving palliative care identification among participants following completion of Module 1,^22^ which was part of the larger trial. This study aims to address two key questions: (1) does participation in CAPACITI improve self-rated comfort and competency in providing a palliative approach to care, and (2) are there differences in outcomes between the facilitated and self-directed study arms?

## Methods

### Study design

This study was a prospective cluster randomized controlled trial (cRCT). Clusters of Canadian primary health care teams were randomized to a facilitated or self-directed learning mode of the CAPACITI program. Details regarding the intervention and the research process are outlined in the published study protocol.^20^ Ethics approval for this research was received from the Hamilton Integrated Research Ethics Board (#13867) (operating protocol is available at https://clinicaltrials.gov/ct2/show/NCT05120154).

### CAPACITI program

The goal of CAPACITI is to impart practical strategies to assist primary care teams in delivering an early palliative approach to care. The program is comprised of three modules: (1) identify and assess, (2) enhance communication skills, and (3) coordinate for ongoing care. Each module consists of four one-hour sessions (12 sessions total). All sessions comprised three core components: (1) clinical education through the provision of expert advice, (2) practical examples via case study integration, and (3) evidence-based tools. Modules were hosted through Moodle, an online learning management system. Further information regarding the content, development, and structure of CAPACITI has been detailed in previous publications.^17,20^

### Participants and recruitment

Study participants included interprofessional primary care participants who had enrolled in the CAPACITI program. Participants were able to participate as members of a primary care team or as sole providers. Our participants were community-based health providers interested in providing palliative care to their patients. Team-based participants had to include at least one prescribing clinician (a physician or nurse practitioner).

We recruited participants through promotional materials, which included direct solicitation and/or email adverts through partner organizations. Teams who were interested in participating in CAPACITI completed a registration form enlisting which members from their team were to be included. Individual members of teams registered on Moodle to enroll. Participants were encouraged to complete Pallium Canada’s LEAP course^7^ before beginning the CAPACITI program to provide clinical knowledge about palliative care.

### Study groups and randomization

The self-directed arm of this study served as the control group. They received access to the CAPACITI virtual session materials. The facilitated arm comprised the intervention group and received the same access to online materials alongside invitations to participate in virtual webinars. One joint webinar was administered per session (four per module, one hour in length) through Zoom for all participants in the intervention group to join. The webinars consisted of a live presentation followed by an open discussion facilitated by a palliative care clinician who responded to questions and shared advice.

The unit of randomization for our study was the team. Randomization to the intervention or control arm occurred separately for each of the three modules using a permuted block design to ensure that group sizes were approximately equal.^23^ The rationale for randomization following each module was so that participants could have an equal opportunity to experience both the facilitated and self-directed arms of the study for their own educational experiences. For Module 1, teams registered in CAPACITI were randomized to the study groups, after they completed the baseline survey. Randomization for Modules 2 and 3 occurred following enrollment in each module. Randomization was stratified through team size and geographical location to balance sub-group characteristics. Randomization was conducted using a computer-generated sequence managed by an independent statistician.

### Data collection and outcomes

The primary study outcome was participants’ perceived comfort with applying palliative care skills, based on their responses to the End-of-Life Professional Caregiver Survey (EPCS).^24^ The secondary outcome was participant-reported competence in applying the elements of a palliative approach to care taught in CAPACITI, measured by the CAPACITI Competencies survey. Outcomes and covariates were collected using online surveys administered to participants through Moodle, both prior to and following completion of each module. Participants who completed the post-survey for a module did not complete the pre-survey for the next module, given the close temporal proximity between modules. We followed the Dillman Tailored Design Method to administer the questionnaire with up to seven follow-up emails to nonresponders.^25^

The EPCS is a validated instrument to assess the palliative and end-of-life care-specific educational needs of multidisciplinary health teams, stated in terms of comfort in applying specified skills.^24^ The survey’s design integrates the eight domains of American national palliative care guidelines and assesses core elements of physician- and nurse-specific end-of-life education curricula.^24,26,27^ Each item is scored on a 5-point Likert scale ranging from 1 (low level of skill) to 5 (highest level of skill). We used a 20-item modified version of the EPCS consisting of two subdomains: effective care delivery (ECD, 8 items), and patient and family-centered communication (12 items).

We developed the Competencies survey based on the CanMEDS framework for improving patient care through enhancing physician training and the topics addressed in the CAPACITI program. The 20 items on the Competencies survey are scored with a 7-point Likert scale, ranging from 1 (low level of confidence) to 7 (highest level of confidence). We pilot tested this survey with 127 multidisciplinary health care providers participating in an earlier iteration of CAPACITI, with no modifications deemed necessary to the survey.^20^

### Data analysis

The complete CAPACITI program (three modules) was delivered in two separate cohorts (November–December 2021 and April–May 2022). This comprised a total of 185 teams, representing 566 individuals who enrolled in CAPACITI. Teams were recruited from 11 provinces or territories across Canada, and the number of members per team ranged from single-provider teams to a maximum of 28 members, with 62.7% of “teams” consisting of a single provider (see Table 1 for participant demographics). Overall, 31.1% of participants were from small teams (1–3 members) and 68.9% from large teams (4+ members). Approximately one-third (33.9%) of allocations did not match the participants’ preferred mode.

**Table 1.** Participant Demographics of Those Completing Module 1 (Including in Main Analysis), *N* = 378

Participant characteristics	Allocation arm		Total (*N* = 378)
	Facilitated (*N* = 169)	Self-directed (*N* = 209)	
Gender			
Female	148 (87.6)	168 (80.4)	316 (83.6)
Male	20 (11.8)	37 (17.7)	57 (15.1)
Gender diverse	1 (0.592)	4 (1.91)	5 (1.32)
Role/profession			
Registered nurse/registered practical nurse	53 (31.4)	65 (31.1)	118 (31.2)
Physician	23 (13.6)	25 (12.0)	48 (12.7)
Paramedic	24 (14.2)	20 (9.57)	44 (11.6)
Allied health	21 (12.4)	18 (8.61)	39 (10.3)
Social worker	14 (8.28)	21 (10.0)	35 (9.26)
Nurse practitioner	18 (10.7)	16 (7.66)	34 (8.99)
Administration	7 (4.14)	20 (9.57)	27 (7.14)
Care coordinator	1 (0.592)	9 (4.3)	10 (2.65)
Pharmacist	4 (2.37)	6 (2.87)	10 (2.65)
Support Worker/aide/attendant	2 (1.18)	4 (1.91)	6 (1.59)
Other non (direct) provider	2 (1.18)	5 (2.39)	7 (1.85)
Former palliative care training			
LEAP	78 (46.2)	89 (42.6)	167 (44.2)
Other	27 (16.0)	35 (16.7)	62 (16.4)
None	64 (37.9)	85 (40.7)	149 (39.4)

We analyzed and stored data using IBM SPSS Statistics (version 29). Data cleaning procedures were conducted to check for missing values and data inconsistencies. The significance level was set at *α* = 0.05 for all statistical tests, and analyses were conducted using two-tailed tests. Results were interpreted to account for effect sizes and confidence intervals (CIs) to assess the magnitude of observed effects.

Standard summary statistics were calculated for the provider and team characteristics, outcomes, and other covariates. Data were analyzed separately for each of the three modules comparing changes in individual paired scores from pre- to post-module, between study groups and overall. We also compared paired outcomes overall across all three modules for participants that completed the full CAPACITI program, in addition to examining the linear trend and overall change in pre- and post-survey summary scores. The following data about covariates, which may have impacted the program’s efficacy were collected and analyzed: (1) team and practitioner characteristics and (2) individual’s preferred learning style (self-directed or facilitated). We recorded participants’ preferred mode of learning (facilitated vs. self-directed), and those who indicated no preference were counted as a match.

The projected sample size was calculated based on the detection of a 0.5-point difference increase on the EPCS based on a standard deviation 1.0 (scale from 1 to 5).^20^ Regarding the cluster design, the correlation between providers within teams was estimated to be 0.15 based on our previous studies^17^ and that each team would have a minimum of four members participating. Given these parameters, 192 providers from 48 teams were calculated as the minimum required sample size (*α* = 0.05, power 80%).

## Results

**FIG. 1.**
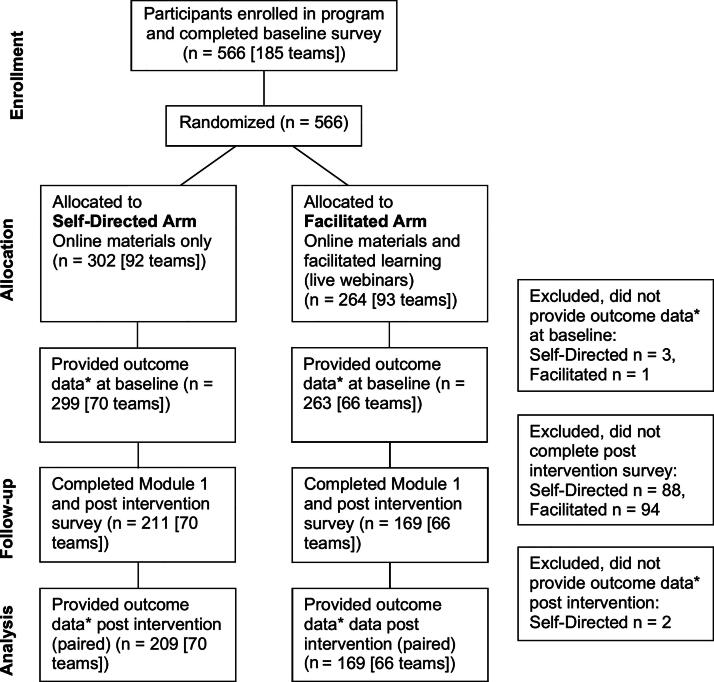
CAPACITI Study Consort Diagram, Module 1. This figure shows the enrollment, allocation to either the self-directed or facilitated study arm, the number of participants at follow-up, and those included in the final analysis (e.g., those who completed the entirety of Module 1). CAPACITI, Community Access to Palliative Care via Interprofessional Teams Improvement.

In total, 378 participants completed Module 1 and are included in our main analysis (see Fig. 1 for the Module 1 consort diagram, and Supplementary Figs. S1 and Figs. S2 for those of Modules 2 and 3). Module 2 and Module 3 were, respectively, completed by 270 (response rate of 70.1%) and 192 (response rate of 71.4%) individuals. Overall, 192 participants completed all three modules, and a total of 374 individuals were lost to follow-up. The characteristics and allocations of participants who were included or excluded from the analysis were similar.

Approximately one-third (31.2%) of paired participants included in the main analysis were nurses. Other represented professions included physicians (12.7%), paramedics (11.6%), allied health workers (10.3%), and nurse practitioners (9.0%). Most included participants had completed Pallium LEAP (44.2%) or had received previous palliative care education (16.4%) prior to enrolling in CAPACITI.

This analysis focuses on secondary outcomes related to self-rated comfort and competency in providing a palliative approach. Significant improvements in self-rated comfort and competency in applying a palliative approach to care were observed following the program, both for the facilitated and self-directed groups. There was no discernible difference between the two delivery modalities.

### Overall pre- versus post-differences

**FIG. 2.**
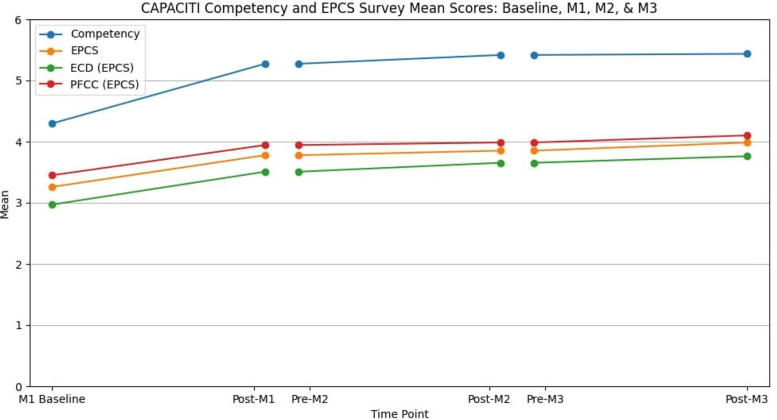
Competency survey and EPCS summary mean scores pre- and post-CAPACITI, for each module. This line graph depicts the pre- and post-mean score gains measured across both surveys, as well as for the two subdomains of the EPCS (ECD and PFCC). Competency is measured on a 7-point scale, and EPCS is measured on a 5-point scale. ECD, effective care delivery EPCS, End-of-Life Professional Caregiver Survey; PFCC, patient and family-centered communication.

From baseline to the completion of Module 1, there were significant gains in self-rated competency (summary means) across both surveys (*p* < 0.001) (pre- to post-Module 1 mean paired difference: Competency = 0.98 [95% CI, 0.82–1.15]; EPCS = 0.49 [95% CI, 0.39–0.59]) including in the subdomains of ECD and patient-family centered communication, as shown in Table 2. Module 1 represents the largest increase in self-rated confidence across the entire CAPACITI program, with post-module increases in self-rated confidence plateauing across Modules 2 and 3. Figure 2 presents a graph of the pre- and post-survey summary means for each module. Moderate pre- to post-improvements were seen for Module 2 (both surveys, *p* = 0.07) and for Module 3, although a significant improvement was only found for the EPCS (*p* < 0.001).

**Table 2.** Paired *t* Tests for Survey Summary Score Means of M1 (*n* = 378), M2 (*n* = 270), M3 (*n* = 192), and Across All Modules (*n* = 192)

Domain	Time point comparison, all paired participants by module	Mean paired difference (SD)	Confidence interval (CI) [95% lowest CI, highest CI]	*p*-Value (two-sided)
Competency	Post-M1–Pre-M1	0.98 (1.49)	[0.82, 1.15]	**<0.001**
	Post-M2–Pre-M2	0.15 (1.22)	[−0.01, 0.31]	**0.07**
	Post-M3–Pre-M3	0.03 (0.57)	[−0.05, 0.12]	**0.46**
	Post-M3–Pre-M1	1.10 (1.01)	[0.95, 1.26]	**<0.001**
EPCS	Post-M1–Pre-M1	0.49 (0.96)	[0.39, 0.59]	**<0.001**
	Post-M2–Pre-M2	0.10 (0.89)	[−0.01, 0.22]	**0.07**
	Post-M3–Pre-M3	0.01 (0.41)	[0.03, 0.16]	**<0.005**
	Post-M3–Pre-M1	0.70 (0.59)	[0.61, 0.79]	**<0.001**
ECD (EPCS)	Post-M1–Pre-M1	0.55 (1.11)	[0.44, 0.67]	**<0.001**
	Post-M2–Pre-M2	0.15 (1.00)	[0.03, 0.28]	**0.02**
	Post-M3–Pre-M3	0.10 (0.53)	[0.02, 0.18]	**0.02**
	Post-M3–Pre-M1	0.78 (.072)	[0.67, 0.89]	**<0.001**
PFCC (EPCS)	Post-M1–Pre-M1	0.45 (0.96)	[0.35, 0.55]	**<0.001**
	Post-M2–Pre-M2	0.06 (0.89)	[−0.05, 0.17]	**0.30**
	Post-M3–Pre-M3	0.09 (0.42)	[0.03, 0.16]	**<0.005**
	Post-M3–Pre-M1	0.63 (0.61)	[0.54, 0.72]	**<0.001**

ECD, effective care delivery EPCS, End-of-Life Professional Caregiver Survey; PFCC, patient and family-centered communication.

Across covariates analyzed in the general linear model (GLM), the only association detected was between improvement on the Competency survey post-Module 1 and larger team size (*p* = 0.019). Location, allocation to one’s preferred mode of learning, previous training, and professional role were not found to be predictive of the study outcomes.

The other analysis of interest in this study was the overall trend in EPCS and Competency survey scores across all three CAPACITI modules for participants who completed the full program (*n* = 192). For this subpopulation, the mean paired summary scores followed a similar trend across modules to that analyzed individually by module, as reported above (Table 2 and Supplementary Fig. S5). Overall, a paired mean gain of 1.1 from baseline to post-Module 3 was calculated for the Competency survey (*p* < 0.001 [95% CI, 0.95–1.26]) and 0.70 for the EPCS (*p* < 0.001 [95% CI, 0.61–0.79]).

### Comparison of facilitated to self-directed groups

Our primary analysis was the paired differences in application of palliative care skills between the facilitated (experimental) and self-directed (control) groups, following completion of CAPACITI Module 1, with the same comparisons each for Module 2 and Module 3 as secondary outcomes. Module 1 was treated as the primary outcome because the skills taught (namely identification) aligned most closely with palliative care competencies. We did not account for the clustering effect of the teams in our analysis due to the large number of single person teams, the small differences between intra- and inter-team correlations on EPCS mean scores (Levene’s Test of Equality of Error Variances, median *p* = 0.15), and our prior analysis finding that clustering had a minimal effect.^17^ Univariate analyses were conducted after completion of each module to determine if a significant difference in outcomes occurred between the groups.

Individual item means pre- and post-Module 1 for the EPCS and the Competency survey were similar between the facilitated and self-directed groups (Supplementary Figs. S3 and Figs. S4), with all items improving. On the EPCS, mean paired pre/post-differences for the groups combined ranged from an increase of 0.3 for “I am comfortable talking with other health care professionals about the care of dying patients” to 0.8 for “I am familiar with palliative care principles and national guidelines”. Similarly, for the Competency survey, mean paired pre/post-differences for the groups combined ranged from an increase of 0.6 for “Making a home visit to the patient when needed” to 1.2 for “Applying evidence-based tools to implement a palliative care approach in your practice.”

Both study groups had similar summary mean scores on the EPCS and the Competency survey at baseline (Table 3). No significant differences were detected between groups following any of the modules on either measure (*p* = 0.09–0.98). While both groups were approximately similar in pre-post-differences by module, the magnitude of improvement occasionally differed between allocation arms. Post-Module 2 findings trended toward the facilitated group having greater improvements from the previous module than the self-directed group across each survey (Competency: 5.38 and 5.28; EPCS: 3.85 and 3.73); however, this difference was not statistically significant (*p* = 0.47 and *p* = 0.2, respectively). This gap closed post-Module 3. An analysis using a GLM found that none of the selected covariates significantly impacted the similarity in the dependent outcomes between groups for any of the modules.

**Table 3.** Allocation Arm and Survey Summary Means at Each Module and Mean Paired Differences

	Facilitated mean (SD)	Self-directed mean (SD)	Mean difference (facilitated–self-directed)	*p*-Value (two-sided)
BASELINE				
Competency	4.30 (1.23)	4.20 (1.30)	0.10	0.48
EPCS	3.24 (0.71)	3.19 (0.78)	0.04	0.60
ECD	2.90 (0.81)	2.90 (0.85)	0.00	0.98
PFCC	3.46 (0.73)	3.38 (0.81)	0.08	0.35
POST-M1				
Competency	5.18 (0.89)	5.25 (0.88)	−0.08	0.44
EPCS	3.71 (0.65)	3.72 (0.63)	−0.01	0.88
ECD	3.44 (0.77)	3.48 (0.75)	−0.04	0.62
PFCC	3.89 (0.65)	3.86 (0.63)	0.03	0.70
POST-M2				
Competency	5.38 (0.85)	5.28 (0.95)	0.09	0.47
EPCS	3.85 (0.64)	3.73 (0.70)	0.11	0.20
ECD	3.62 (0.76)	3.56 (0.79)	0.04	0.67
PFCC	3.99 (0.62)	3.85 (0.70)	0.13	0.09
POST-M3				
Competency	5.42 (0.89)	5.41 (0.82)	0.02	0.78
EPCS	3.93 (0.65)	3.95 (0.60)	−0.01	0.82
ECD	3.66 (0.76)	3.78 (0.72)	−0.11	0.13
PFCC	4.07 (0.62)	4.06 (0.56)	0.02	0.78

ECD, effective care delivery EPCS, End-of-Life Professional Caregiver Survey; PFCC, patient and family-centered communication.

## Discussion

Our randomized controlled trial yielded two key findings: (1) self-assessed comfort and competency in providing an early palliative care approach improved significantly in both study arms, with the largest gains occurring between baseline and post-Module 1, and (2) no significant difference was observed between the two study arms.

Facilitated sessions did not provide added benefit over self-directed learning. This assumption, based on virtual teaching best practices emphasizing live discussion-based webinars^28^ aligns with studies suggesting facilitation type is less impactful than program content.^29–31^ A review of palliative care training programs^32^ identified only two studies comparing facilitated and nonfacilitated interventions, both focused on nursing.^33,34^ These studies showed instructor-led facilitation improved confidence and problem-solving skills compared with self-directed formats but did not examine interprofessional primary care providers as in our study. A scoping review of serious illness communication training noted heterogeneity prevents identifying a superior educational format.^35^

Several factors may explain why both groups had similar outcomes in the CAPACITI program. Facilitators did not offer tailored advice beyond webinar content, which supports active learning.^36^ Participants may have been motivated to engage regardless of facilitation, as evidenced by high baseline proficiency. The study’s timing during the COVID-19 pandemic likely increased comfort with virtual, self-directed learning.^36,37^

The CAPACITI program is grounded in adult learning practices, case-based learning, and integration of real-world scenarios relevant to primary care. These features may have contributed to participants’ perceived engagement and knowledge retention. Module 1, which saw the largest gains, focused on patient identification and early assessment, which are foundational areas of palliative care education. The stepwise structure of the program and clear learning objectives may have further bolstered participant confidence in applying knowledge to practice.

Our study is among the first randomized trials to measure perceived palliative care competency after virtual training.^32^ Across all modules, CAPACITI participants reported significant increases in confidence, particularly after Module 1, which focused on identifying and assessing patients for a palliative approach.^17^ All modules contributed to increased confidence, highlighting the program’s value in informing clinical practice.

The findings demonstrate that even in the absence of facilitation, self-directed virtual programs can yield meaningful improvements in clinical competency. This has implications for other health care areas where accessibility, scalability, and efficiency are critical. Lessons from this study may inform training initiatives in areas such as chronic disease management or geriatric care, particularly where programs are grounded in content-relevant content and real-world applicability. Future research should investigate the optimal role of facilitation in developing complex clinical skills, such as communication.

### Limitations

A core limitation of this study is that the outcomes from both surveys were self-reported, thus creating the potential exaggeration of post-intervention behavioral changes. From baseline to the end of Module 3, approximately one-third of participants were lost to follow-up after each module. CAPACITI was designed as three distinct modules that could be taken independently, and some participants may not have enrolled in subsequent modules. This attrition rate is reflective of those of similar educational training interventions.^32^ We could not compare study groups across the entire duration of the program because participants were re-randomized to either study arm for each module.

## Conclusion

Our findings show that participation in CAPACITI improved self-assessed palliative care skills for providers following completion of each of the three modules, with no discernible difference between facilitated and self-directed groups. CAPACITI demonstrates that a relatively large, intensive, and feasible training program can be implemented virtually across diverse care settings. These results support the broader application of structured, scalable educational interventions in primary care, particularly those grounded in practical content and adult learning principles.

## Data Availability

De-identified data are available from the corresponding author upon reasonable request.

## Patient and Public Involvement

Patients or the public were not involved in the design of this research.
