# Supplementary material for: Facilitated Versus Self-Directed Educational Modalities in Palliative Care Training: A Randomized Controlled Trial of the CAPACITI Intervention
Source: Palliat Med Rep. 2025 Jun 17;6(1):365–73. doi: 10.1089/pmr.2025.0010 (PMC12410323; doi:10.1089/pmr.2025.0010)
Supplement: Supplementary Figures [file pmr.2025.0010_supp_figures.docx]

**Supplemental Figures**

**Figure S1: CAPACITI Study Consort Diagram Module 2**

Participants enrolled in program and completed M2 baseline survey (n = 387 [136 teams])

# Enrollment

Randomized (n = 387)

Allocated to

**Facilitated Arm** Online materials and facilitated learning (live webinars)

(n = 205 [70 teams])

Follow-up

Allocation

Allocated to

**Self-Directed Arm** Online materials only

(n = 182 [66 teams])

Excluded, did not provide outcome data* at baseline:

Self-Directed n = 0, Facilitated n = 2

Provided outcome data* at baseline (n = 203 [70 teams])

Provided outcome data* at baseline (n = 182 [66 teams])

Excluded, did not complete post intervention survey: Self-Directed n = 52, Facilitated n = 59

Completed Module 2 and post intervention survey (n = 144 [54 teams])

Completed Module 2 and post intervention survey (n = 130 [58 teams])

Excluded, did not provide outcome data* post intervention:

Self-Directed n = 1, Facilitated n = 3

# Analysis

Provided outcome data* data post intervention (paired) (n = 141 [54 teams])

Provided outcome data* post intervention (paired) (n = 129 [58 teams])

**Figure S2: CAPACITI Study Consort Diagram Module 3**

Participants enrolled in program and completed M3 baseline survey (n = 269 [112 teams])

# Enrollment

Randomized (n = 269)

Allocated to

**Facilitated Arm** Online materials and facilitated learning (live webinars)

(n = 126 [57 teams])

Follow-up

Allocation

Allocated to

**Self-Directed Arm** Online materials only

(n = 143 [55 teams])

Excluded, did not provide outcome data* at baseline:

Self-Directed n = 0, Facilitated n = 0

Provided outcome data* at baseline (n = 126 [57 teams])

Provided outcome data* at baseline (n = 143 [55 teams])

Excluded, did not complete post intervention survey: Self-Directed n = 35, Facilitated n = 36

Completed Module 2 and post intervention survey (n = 90 [45 teams])

Completed Module 2 and post intervention survey (n = 108 [47 teams])

Excluded, did not provide outcome data* post intervention:

Self-Directed n = 3, Facilitated n = 3

# Analysis

Provided outcome data* data post intervention (paired) (n = 87 [44 teams])

Provided outcome data* post intervention (paired) (n = 105 [47 teams])

**Figure S3: Pre-post Module 1 scores by EPCS survey item**

|  | **Facilitated (n=162)** | | | **Self-Directed (n=193)** | | | **Total (n=355)** | | |
| --- | --- | --- | --- | --- | --- | --- | --- | --- | --- |
|  | Pre-Mean (SD) | Post-Mean (SD) | Mean Paired Difference | Pre-Mean (SD) | Post-Mean (SD) | Mean Paired Difference | Pre-Mean (SD) | Post-Mean (SD) | Mean Paired Difference |
| 1. I am comfortable helping families to accept a poor prognosis | 3.29 (0.98) | 3.71 (0.85) | 0.43 | 3.21 (1.05) | 3.61 (0.98) | 0.36 | 3.25 (1.02) | 3.66 (0.92) | 0.39 |
| 2. I am able to set goals for care with patients and families | 3.54 (1.00) | 3.92 (0.87) | 0.39 | 3.29 (1.04) | 3.87 (0.89) | 0.58 | 3.40 (1.03) | 3.89 (0.88) | 0.50 |
| 3. I am comfortable talking to patients and families about personal choice and self-determination | 3.65 (1.10) | 4.07 (0.89) | 0.42 | 3.56 (1.05) | 4.04 (0.86) | 0.46 | 3.60 (1.07) | 4.06 (0.87) | 0.44 |
| 4. I am comfortable starting and participating in discussions about code status | 3.35 (1.26) | 3.99 (1.02) | 0.66 | 3.42 (1.20) | 3.86 (1.03) | 0.37 | 3.38 (1.23) | 3.91 (1.02) | 0.51 |
| 5. I can assist family members and others through the grieving process | 3.4 (1.04) | 3.69 (1.00) | 0.28 | 3.24 (1.14) | 3.72 (0.92) | 0.45 | 3.31 (1.10) | 3.70 (0.96) | 0.37 |
| 6. I am able to document the needs and interventions of my patients | 4.02 (0.88) | 4.28 (0.79) | 0.24 | 3.83 (0.99) | 4.30 (0.77) | 0.47 | 3.92 (0.95) | 4.29 (0.78) | 0.37 |
| 7. I am comfortable talking with other health care professionals about the care of dying patients | 4.06 (0.88) | 4.30 (0.70) | 0.23 | 3.95 (0.91) | 4.32 (0.81) | 0.35 | 4.00 (0.90) | 4.31 (0.76) | 0.30 |
| 8. I am comfortable helping to resolve difficult family conflicts about end-of-life care | 2.75 (1.08) | 3.33 (1.10) | 0.58 | 2.71 (1.22) | 3.33 (1.08) | 0.60 | 2.72 (1.16) | 3.33 (1.09) | 0.59 |
| 9. I can recognize impending death (physical changes) | 3.61 (1.13) | 4.10 (0.92) | 0.47 | 3.57 (1.12) | 3.93 (0.94) | 0.35 | 3.59 (1.12) | 4.01 (0.94) | 0.41 |
| 10. I know how to use non-drug therapies in management of patients’ symptoms | 2.88 (1.03) | 3.56 (0.98) | 0.67 | 2.98 (1.17) | 3.58 (0.95) | 0.58 | 2.93 (1.11) | 3.57 (0.96) | 0.62 |
| 11. I am able to address patients’ and family members’ fears of getting addicted to pain medications | 3.29 (1.08) | 3.93 (0.93) | 0.63 | 3.33 (1.15) | 3.83 (1.01) | 0.48 | 3.31 (1.12) | 3.88 (0.97) | 0.55 |
| 12. I encourage patients and families to complete advanced care planning | 3.61 (1.18) | 3.97 (0.99) | 0.37 | 3.59 (1.13) | 4.03 (0.94) | 0.42 | 3.60 (1.15) | 4.01 (0.96) | 0.40 |
| 13. I can recognize when patients are appropriate for referral to hospice | 3.02 (1.22) | 3.74 (1.01) | 0.72 | 3.08 (1.19) | 3.66 (0.95) | 0.52 | 3.05 (1.20) | 3.70 (0.98) | 0.61 |
| 14. I am familiar with palliative care principles and national guidelines | 2.73 (1.03) | 3.45 (1.01) | 0.69 | 2.64 (1.10) | 3.46 (0.99) | 0.81 | 2.68 (1.07) | 3.45 (0.99) | 0.76 |
| 15. I am effective at helping patients and families navigate the health care system | 3.31 (0.91) | 3.75 (0.95) | 0.46 | 3.26 (1.01) | 3.80 (0.86) | 0.51 | 3.28 (0.96) | 3.78 (0.90) | 0.49 |
| 16. I am familiar with the services that residential or volunteer hospice providers deliver | 2.56 (1.20) | 3.15 (1.13) | 0.56 | 2.67 (1.21) | 3.29 (1.10) | 0.67 | 2.62 (1.21) | 3.23 (1.12) | 0.62 |
| 17. I am effective at helping to maintain continuity across care settings | 3.07 (0.98) | 3.55 (0.91) | 0.52 | 3.15 (1.08) | 3.68 (0.88) | 0.55 | 3.11 (1.03) | 3.62 (0.89) | 0.53 |
| 18. I feel confident addressing requests for medical assistance in dying | 2.81 (1.33) | 3.15 (1.25) | 0.30 | 2.58 (1.38) | 3.20 (1.23) | 0.64 | 2.69 (1.36) | 3.18 (1.24) | 0.48 |
| 19. I have personal resources to help meet my needs when working with dying patients and families | 3.04 (1.17) | 3.49 (1.09) | 0.49 | 2.89 (1.22) | 3.53 (1.10) | 0.62 | 2.96 (1.19) | 3.51 (1.09) | 0.56 |
| 20. I feel that my workplace provides resources to support staff who care for dying patients | 2.66 (1.22) | 3.23 (1.18) | 0.55 | 2.93 (1.19) | 3.18 (1.23) | 0.21 | 2.81 (1.21) | 3.20 (1.20) | 0.36 |
| All Items | 3.23 (1.08) | 3.72 (0.98) | 0.48 | 3.19 (1.13) | 3.71 (0.98) | 0.5 | 3.21 (1.0) | 3.71 (0.98) | 0.49 |

**Figure S4: Pre-post Module 1 scores by Competency survey item**

|  | **Facilitated (n=155)** | | | **Self-Directed (n=185)** | | | **Total (n=340)** | | |
| --- | --- | --- | --- | --- | --- | --- | --- | --- | --- |
|  | Pre-Mean (SD) | Post-Mean (SD) | Mean Paired Difference | Pre-Mean (SD) | Post-Mean (SD) | Mean Paired Difference | Pre-Mean (SD) | Post-Mean (SD) | Mean Paired Difference |
| 1. Identifying all patients requiring palliative care at end of life | 4.49 (1.61) | 5.46 (1.12) | 0.92 | 4.47 (1.63) | 5.50 (1.05) | 1.10 | 4.48 (1.62) | 5.48 (1.08) | 1.01 |
| 2. Identifying all patients requiring palliative care following diagnosis of a progressive life-limiting disease | 4.33 (1.60) | 5.34 (1.20) | 0.96 | 4.30 (1.60) | 5.34 (1.13) | 1.05 | 4.31 (1.60) | 5.34 (1.16) | 1.01 |
| 3. Having an open, honest conversation with patients about their illness trajectory | 4.23 (1.58) | 5.21 (1.20) | 0.95 | 4.11 (1.73) | 5.16 (1.22) | 1.06 | 4.16 (1.66) | 5.18 (1.21) | 1.01 |
| 4. Ongoing assessment of patients’ palliative care needs | 4.40 (1.41) | 5.25 (1.00) | 0.82 | 4.27 (1.65) | 5.38 (1.02) | 1.14 | 4.33 (1.54) | 5.32 (1.01) | 0.99 |
| 5. Ongoing management of patients’ palliative care needs | 4.31 (1.47) | 5.03 (1.09) | 0.72 | 4.10 (1.65) | 5.18 (1.08) | 1.08 | 4.20 (1.57) | 5.11 (1.09) | 0.91 |
| 6. Care planning with the patient following diagnosis of a progressive life-limiting disease | 4.09 (1.54) | 4.98 (1.23) | 0.89 | 3.94 (1.60) | 5.09 (1.22) | 1.16 | 4.01 (1.57) | 5.04 (1.22) | 1.03 |
| 7. Care planning with the family caregiver following diagnosis of a progressive life-limiting disease | 4.07 (1.53) | 5.01 (1.22) | 0.92 | 3.95 (1.62) | 5.23 (1.13) | 1.29 | 4.01 (1.58) | 5.13 (1.17) | 1.12 |
| 8. Care planning with the patient during a “crisis” (e.g., rapid exacerbation of symptoms) | 4.04 (1.67) | 5.00 (1.30) | 0.99 | 3.93 (1.64) | 5.09 (1.31) | 1.15 | 3.98 (1.66) | 5.05 (1.31) | 1.08 |
| 9. Care planning with the family caregiver during a “crisis” | 4.07 (1.58) | 4.95 (1.21) | 0.93 | 3.96 (1.65) | 5.08 (1.27) | 1.09 | 4.01 (1.62) | 5.02 (1.24) | 1.02 |
| 10. Care planning with the patient during the end of life stage | 4.32 (1.62) | 5.15 (1.27) | 0.86 | 4.20 (1.68) | 5.12 (1.32) | 0.96 | 4.26 (1.65) | 5.13 (1.29) | 0.92 |
| 11. Care planning with the family caregiver during the end of life stage | 4.30 (1.63) | 5.13 (1.27) | 0.89 | 4.16 (1.68) | 5.20 (1.28) | 1.07 | 4.23 (1.66) | 5.17 (1.27) | 0.99 |
| 12. Making a home visit to the patient when needed | 5.26 (1.72) | 5.72 (1.38) | 0.28 | 4.95 (1.79) | 5.82 (1.34) | 0.86 | 5.09 (1.76) | 5.77 (1.36) | 0.60 |
| 13. Coordinating care planning with the patient’s providers (e.g., pharmacist, home care nurse) who are outside of your team | 4.78 (1.45) | 5.69 (1.05) | 0.90 | 4.72 (1.62) | 5.60 (1.18) | 0.81 | 4.75 (1.54) | 5.64 (1.12) | 0.85 |
| 14. Engaging with a Palliative Care Specialist regarding the patient’s care | 4.65 (1.56) | 5.51 (1.15) | 0.78 | 4.71 (1.57) | 5.42 (1.19) | 0.72 | 4.68 (1.57) | 5.46 (1.17) | 0.75 |
| 15. Connecting directly with Disease Specialists regarding the patient’s care | 4.18 (1.60) | 5.15 (1.18) | 0.96 | 4.23 (1.68) | 5.05 (1.36) | 0.76 | 4.21 (1.64) | 5.10 (1.28) | 0.85 |
| 16. Keeping abreast of all local community support services relevant to palliative care | 4.08 (1.59) | 4.96 (1.33) | 0.84 | 4.03 (1.66) | 5.07 (1.31) | 1.08 | 4.06 (1.63) | 5.02 (1.32) | 0.97 |
| 17. Ongoing reflection and strategizing on ways to strengthen how your team works with each other | 4.24 (1.44) | 5.21 (1.06) | 0.97 | 4.32 (!.44) | 5.28 (1.01) | 0.92 | 4.28 (1.44) | 5.25 (1.04) | 0.95 |
| 18. Ongoing reflection and strategizing on ways to strengthen how your team works with external providers | 4.06 (1.42) | 4.98 (1.19) | 0.89 | 4.10 (1.48) | 5.09 (1.11) | 0.97 | 4.08 (1.45) | 5.04 (1.14) | 0.93 |
| 19. Applying evidence based tools to implement a palliative care approach in your practice (e.g. tools to identify/monitor/assess patients) | 4.05 (1.49) | 5.25 (1.08) | 1.22 | 3.97 (1.52) | 5.22 (1.10) | 1.25 | 4.01 (1.50) | 5.23 (1.09) | 1.24 |
| 20. Using data from your health information system (e.g., EMR or paper) to optimize a palliative care approach | 3.83 (1.67) | 4.94 (1.32) | 1.18 | 3.92 (1.65) | 4.96 (1.36) | 1.04 | 3.88 (1.66) | 4.95 (1.34) | 1.10 |
| All Items | 4.29 (1.56) | 5.20 (1.19) | 0.89 | 4.22 (1.63) | 5.24 (1.2) | 1.03 | 4.25 (1.6) | 5.22 (1.2) | 0.97 |

**Figure S5: Line graph depicting survey mean score gains following each module**
